# The psychological functions of music listening

**DOI:** 10.3389/fpsyg.2013.00511

**Published:** 2013-08-13

**Authors:** Thomas Schäfer, Peter Sedlmeier, Christine Städtler, David Huron

**Affiliations:** ^1^Department of Psychology, Chemnitz University of TechnologyChemnitz, Germany; ^2^School of Music, Cognitive and Systematic Musicology Laboratory, Ohio State UniversityColumbus, OH, USA

**Keywords:** music, functions of music, self-awareness, social relatedness, arousal regulation, mood regulation

## Abstract

Why do people listen to music? Over the past several decades, scholars have proposed numerous functions that listening to music might fulfill. However, different theoretical approaches, different methods, and different samples have left a heterogeneous picture regarding the number and nature of musical functions. Moreover, there remains no agreement about the underlying dimensions of these functions. Part one of the paper reviews the research contributions that have explicitly referred to musical functions. It is concluded that a comprehensive investigation addressing the basic dimensions underlying the plethora of functions of music listening is warranted. Part two of the paper presents an empirical investigation of hundreds of functions that could be extracted from the reviewed contributions. These functions were distilled to 129 non-redundant functions that were then rated by 834 respondents. Principal component analysis suggested three distinct underlying dimensions: People listen to music to *regulate arousal and mood*, to achieve *self-awareness*, and as an expression of *social relatedness*. The first and second dimensions were judged to be much more important than the third—a result that contrasts with the idea that music has evolved primarily as a means for social cohesion and communication. The implications of these results are discussed in light of theories on the origin and the functionality of music listening and also for the application of musical stimuli in all areas of psychology and for research in music cognition.

## Introduction

Music listening is one of the most enigmatic of human behaviors. Most common behaviors have a recognizable utility that can be plausibly traced to the practical motives of survival and procreation. Moreover, in the array of seemingly odd behaviors, few behaviors match music for commandeering so much time, energy, and money. Music listening is one of the most popular leisure activities. Music is a ubiquitous companion to people's everyday lives.

The enthusiasm for music is not a recent development. Recognizably musical activities appear to have been present in every known culture on earth, with ancient roots extending back 250,000 years or more (see Zatorre and Peretz, 2001). The ubiquity and antiquity of music has inspired considerable speculation regarding its origin and function.

Throughout history, scholars of various stripes have pondered the nature of music. Philosophers, psychologists, anthropologists, musicologists, and neuroscientists have proposed a number of theories concerning the origin and purpose of music and some have pursued scientific approaches to investigating them (e.g., Fitch, 2006; Peretz, 2006; Levitin, 2007; Schäfer and Sedlmeier, 2010).

The origin of music is shrouded in prehistory. There is little physical evidence—like stone carvings or fossilized footprints—that might provide clues to music's past. Necessarily, hypotheses concerning the original functions of music will remain speculative. Nevertheless, there are a number of plausible and interesting conjectures that offer useful starting-points for investigating the functions of music.

A promising approach to the question of music's origins focuses on how music is used—that is, it's various functions. In fact, many scholars have endeavored to enumerate various musical functions (see below). The assumption is that the function(s) that music is presumed to have served in the past would be echoed in at least one of the functions that music serves today. Of course, how music is used today need have no relationship with music's function(s) in the remote past. Nevertheless, evidence from modern listeners might provide useful clues pertinent to theorizing about origins.

In proposing various musical functions, not all scholars have related these functions to music's presumed evolutionary roots. For many scholars, the motivation has been simply to identify the multiple ways in which music is used in everyday lives (e.g., Chamorro-Premuzic and Furnham, 2007; Boer, 2009; Lonsdale and North, 2011; Packer and Ballantyne, 2011). Empirical studies of musical functions have been very heterogeneous. Some studies were motivated by questions related to development. Many related to social identity. Others were motivated by cognitive psychology, aesthetics, cultural psychology, or personality psychology. In addition, studies differed according to the target population. While some studies attempted to assemble representative samples of listeners, others explicitly focused on specific populations such as adolescents. Most studies rely on convenient samples of students. Consequently, the existing literature is something of a hodgepodge.

The aim of the present study is to use the extant literature as a point of departure for a fresh re-appraisal of possible musical functions. In Part 1 of our study, we summarize the results of an extensive literature survey concerning the possible functions of music. Specifically, we identified and skimmed hundreds of publications that explicitly suggest various functions, uses, or benefits for music. We provide separate overviews for the empirical literatures and the theoretical literatures. This survey resulted in just over 500 proposed musical functions. We do not refer to each of the identified publications but concentrate on the ones that have identified either more than one single function of music listening or a single unique function that is not captured in any other publication. In Part 2, we present the results of an empirical study whose purpose was to distill—using principal components analysis (PCA)—the many proposed functions of music listening. To anticipate our results, we will see that PCA suggests three main dimensions that can account for much of the shared variance in the proposed musical functions.

## Review of the research on the functions of music

Discussions and speculations regarding the functions of music listening can be found in both theoretical literature concerning music as well as in empirical studies of music. Below, we offer a review of both literatures. The contents of the reviews are summarized in Tables A1, A2. Table A1 provides an overview of theoretical proposals regarding musical function, whereas Table A2 provides an overview of empirical studies regarding musical function. Together, the two tables provide a broad inventory of potential functions for music.

### Theoretical approaches

Many scholars have discussed potential functions of music exclusively from a theoretical point of view. The most prominent of these approaches or theories are the ones that make explicit evolutionary claims. However, there are also other, non-evolutionary approaches such as *experimental aesthetics* or the *uses-and-gratifications* approach. Functions of music were derived deductively from these approaches and theories. In addition, in the literature, one commonly finds lists or collections of functions that music can have. Most of these lists are the result of literature searches; in other cases authors provide no clear explanation for how they came up with the functions they list. Given the aim of assembling a comprehensive list, all works are included in our summary.

#### Functions of music as they derive from specific approaches or theories

***Evolutionary approaches.*** Evolutionary discussions of music can already be found in the writings of Darwin. Darwin discussed some possibilities but felt there was no satisfactory solution to music's origins (Darwin, 1871, 1872). His intellectual heirs have been less cautious. Miller (2000), for instance, has argued that music making is a reasonable index of biological fitness, and so a manifestation of sexual selection—analogous to the peacock's tail. Anyone who can afford the biological luxury of making music must be strong and healthy. Thus, music would offer an honest social signal of physiological fitness.

Another line of theorizing refers to music as a means of social and emotional communication. For example, Panksepp and Bernatzky (2002, p. 139) argued that

in social creatures like ourselves, whose ancestors lived in arboreal environments where sound was one of the most effective ways to coordinate cohesive group activities, reinforce social bonds, resolve animosities, and to establish stable hierarchies of submission and dominance, there could have been a premium on being able to communicate shades of emotional meaning by the melodic character (prosody) of emitted sounds.

A similar idea is that music contributes to social cohesion and thereby increases the effectiveness of group action. Work and war songs, lullabies, and national anthems have bound together families, groups, or whole nations. Relatedly, music may provide a means to reduce social stress and temper aggression in others. The idea that music may function as a social cement has many proponents (see Huron, 2001; Mithen, 2006; Bicknell, 2007).

A novel evolutionary theory is offered by Falk (2004a,b) who has proposed that music arose from humming or singing intended to maintain infant-mother attachment. Falk's “putting-down-the-baby hypothesis” suggests that mothers would have profited from putting down their infants in order to make their hands free for other activities. Humming or singing consequently arose as a consoling signal indicating caretaker proximity in the absence of physical touch.

Another interesting conjecture relates music to human anxiety related to death, and the consequent quest for meaning. Dissanayake (2009), for example, has argued that humans have used music to help cope with awareness of life's transitoriness. In a manner similar to religious beliefs about the hereafter or a higher transcendental purpose, music can help assuage human anxiety concerning mortality (see, e.g., Newberg et al., 2001). Neurophysiological studies regarding music-induced chills can be interpreted as congruent with this conjecture. For example, music-induced chills produce reduced activity in brain structures associated with anxiety (Blood and Zatorre, 2001).

Related ideas stress the role music plays in feelings of transcendence. For example, (Frith, 1996, p. 275) has noted that: “We all hear the music we like as something special, as something that defies the mundane, takes us “out of ourselves,” puts us somewhere else.” Thus, music may provide a means of escape. The experience of flow states (Nakamura and Csikszentmihalyi, 2009), peaks (Maslow, 1968), and chills (Panksepp, 1995), which are often evoked by music listening, might similarly be interpreted as forms of transcendence or escapism (see also Fachner, 2008).

More generally, Schubert (2009) has argued that the fundamental function of music is its potential to produce pleasure in the listener (and in the performer, as well). All other functions may be considered subordinate to music's pleasure-producing capacity. Relatedly, music might have emerged as a safe form of time-passing—analogous to the sleeping behaviors found among many predators. As humans became more effective hunters, music might have emerged merely as an entertaining and innocuous way to pass time during waking hours (see Huron, 2001).

The above theories each stress a single account of music's origins. In addition, there are mixed theories that posit a constellation of several concurrent functions. Anthropological accounts of music often refer to multiple social and cultural benefits arising from music. Merriam (1964) provides a seminal example. In his book, *The anthropology of music*, Merriam proposed 10 social functions music can serve (e.g., emotional expression, communication, and symbolic representation). Merriam's work has had a lasting influence among music scholars, but also led many scholars to focus exclusively on the social functions of music. Following in the tradition of Merriam, Dissanayake (2006) proposed six social functions of ritual music (such as display of resources, control, and channeling of individual aggression, and the facilitation of courtship).

***Non-evolutionary approaches.*** Many scholars have steered clear of evolutionary speculation about music, and have instead focused on the ways in which people use music in their everyday lives today. A prominent approach is the “uses-and-gratifications” approach (e.g., Arnett, 1995). This approach focuses on the needs and concerns of the listeners and tries to explain how people actively select and use media such as music to serve these needs and concerns. Arnett (1995) provides a list of potential uses of music such as entertainment, identity formation, sensation seeking, or culture identification.

Another line of research is “experimental aesthetics” whose proponents investigate the subjective experience of beauty (both artificial or natural), and the ensuing experience of pleasure. For example, in discussing the “recent work in experimental aesthetics,” Bullough (1921) distinguished several types of listeners and pointed to the fact that music can be used to activate associations, memories, experiences, moods, and emotions.

By way of summary, many musical functions have been proposed in the research literature. Evolutionary speculations have tended to focus on single-source causes such as music as an indicator of biological fitness, music as a means for social and emotional communication, music as social glue, music as a way of facilitating caretaker mobility, music as a means of tempering anxiety about mortality, music as escapism or transcendental meaning, music as a source of pleasure, and music as a means for passing time. Other accounts have posited multiple concurrent functions such as the plethora of social and cultural functions of music found in anthropological writings about music. Non-evolutionary approaches are evident in the uses-and-gratifications approach—which revealed a large number of functions that can be summarized as cognitive, emotional, social, and physiological functions—and the experimental aesthetics approach, whose proposed functions can similarly be summarized as cognitive and emotional functions.

#### Functions of music as they derive from literature research

As noted, many publications posit musical functions without providing a clear connection to any theory. Most of these works are just collections of functions of music from the literature. Not least, there are also accounts of such collections where it remained unclear how the author(s) came up with the functions contained. Some of these works refer to only one single function of music—most often because this functional aspect was investigated not with the focus on music but with a focus on other psychological phenomena. Yet other works list extensive collections of purported musical functions.

Works that refer to only one single functional aspect of music include possible therapeutic functions for music in clinical settings (Cook, 1986; Frohne-Hagemann and Pleß-Adamczyk, 2005), the use of music for symbolic exclusion in political terms (Bryson, 1996), the syntactic, semantic, and mediatizing use of film music (Maas, 1993), and the use of music to manage physiological arousal (Bartlett, 1996).

The vast majority of publications identify several possible musical functions, most of which—as stated above—are clearly focused on social aspects. Several comprehensive collections have been assembled, such as those by Baacke (1984), Gregory (1997), Ruud (1997), Roberts and Christenson (2001), Engh (2006), and Laiho (2004). Most of these studies identified a very large number of potential functions of music.

By way of summary, there exists a long tradition of theorizing about the potential functions of music. Although some of these theories have been deduced from a prior theoretical framework, none was the result of empirical testing or exploratory data-gathering. In the ensuing section, we turn to consider empirically-oriented research regarding the number and nature of potential musical functions.

### Empirical investigations

A number of studies have approached the functions of music from an empirical perspective. Two main approaches might be distinguished. In the first approach, the research aim is to uncover or document actual musical functioning. That is, the research aims to observe or identify one or more ways in which music is used in daily life. In the second approach, the research goal is to infer the structure or pattern underlying the use of music. That is, the research aims to uncover potential basic or fundamental dimensions implied by the multiple functions of music. This is mostly done using PCA or factor analyses or cluster analyses that reduce a large number of functions to only a few basic dimensions. In some cases, the analyses are run exploratively whereas in other cases, they are run in a confirmatory way, that is—with a predefined number of dimensions. The empirical studies can be categorized according to several criteria (see Table A2). However, when discussing some of the most important works here, we will separate studies where respondents were asked for the functions of music in open surveys from studies where the authors provided their own collections of functions, based on either literature research or face validity.

#### Surveys about the functions music can have

A number of studies have attempted to chronicle the broad range of musical functions. Most of these studies employed surveys in which people were asked to identify the ways in which they make use of music in their lives. In some studies, expert interviews were conducted in order to identify possible functions. Table A2 provides a summary of all the pertinent studies including their collections of functions and—where applicable—their derived underlying dimensions. We will restrict our ensuing remarks to the largest and most comprehensive studies.

Chamorro-Premuzic and Furnham (2007) identified 15 functions of music among students and subsequently ran focus groups from which they distilled three distinct dimensions: emotional use, rational use, and background use. Some of the largest surveys have been carried out by Boer (2009). She interviewed more than a thousand young people in different countries and assembled a comprehensive collection of musical functions. Using factor analysis, she found 10 underlying dimensions: emotion, friends, family, venting, background, dancing, focus, values, politic, and culture. (Lonsdale and North, 2011, Study 1) pursued a uses-and-gratifications approach. They identified 30 musical uses that could be reduced to six distinct dimensions. In a related study employing a larger sample, the same authors came up with eight distinct dimensions: identity, positive and negative mood management, reminiscing, diversion, arousal, surveillance, and social interaction (Lonsdale and North, 2011, Study 4). When interviewing older participants, Hays and Minichiello (2005) qualitatively identified six dimensions: linking, life events, sharing and connecting, wellbeing, therapeutic benefits, escapism, and spirituality.

The various surveys and interview studies clearly diverge with regard to the number of different musical functions. Similarly, the various cluster and factor analyses often end up producing different numbers of distinct dimensions. Nevertheless, the results are often quite similar. On a very broad level, there are four categories that appear consistently: social functions, emotional functions, cognitive or self-related functions, and physiological or arousal-related functions (see also Hargreaves and North, 1999; Schäfer and Sedlmeier, 2009, 2010).

#### Empirical studies using predefined collections of functions of music

Apart from the open-ended surveys and interview methods, a number of studies investigating musical functions begin with researcher-defined collections or even categories/dimensions. Some of these predefined collections or categories/dimensions were simply borrowed from the existing published research, whereas others were derived from specific theoretical perspectives.

***Empirical studies on functions of music emerging from specific theoretical approaches.*** Some of the above mentioned theoretical approaches to the functionality of music have been investigated in empirical studies. Boehnke and Münch (2003) developed a model of the relationship of adolescents' development, music, and media use. They proposed seven functions of music that relate to the developmental issues of young people (such as peer group integration, physical maturation, or identity development). In two studies with a large number of participants, Lonsdale and North (2011) applied the model of media gratification (from McQuail et al., 1972) and used a collection of 30 functions of music they assembled from literature research and interviews. In both studies, they ran factor analyses—reducing the number of functions to six dimensions and eight dimensions, respectively. Lehmann (1994) developed a situations-functions-preference model and proposed that music preferences emerge from the successful use of music to serve specific functions for the listener, depending on the current situation. Lehmann identified 68 ways in which people use music, from which he was able to reduce them to 15 music reception strategies (Rezeptionsweisen) such as compensation/escapism, relaxation, and identification. Misenhelter and Kaiser (2008) adopted Merriam's (1964) anthropological approach and attempted to identify the functions of music in the context of music education. They surveyed teachers and students and found six basic functions that were quite similar to the ones proposed by Merriam (1964). Wells and Hakanen (1997) adopted Zillmann's (1988a,b) mood management theory and identified four types of users regarding the emotional functions of music: mainstream, music lover, indifferent, and heavy rockers.

***Empirical studies on functions of music emerging from literature research.*** A number of studies have made use of predefined musical functions borrowed from the existing research literature. The significance of these functions and/or their potential underlying structure has then been empirically investigated using different samples. As mentioned, not all of those studies tried to assemble an exhaustive collection of musical functions in order to produce a comprehensive picture of the functions of music; but many studies were focused on specific aspects such as the emotional, cognitive, or social functions of music.

Schäfer and Sedlmeier (2009) collected 17 functions of music from the literature and found functions related to the management of mood and arousal as well as self-related functions to be the ones that people highly ascribe to their favorite music. Tarrant et al. (2000) used a collection of 10 functions of music from the literature and factor analyzed them resulting in three distinct dimensions of music use: self-related, emotional, and social.

Sun and Lull (1986) collected 18 functions of music videos and were able to reduce them to four dimensions: social learning, passing time, escapism/mood, and social interaction. Melton and Galician (1987) identified 15 functions of radio music and music videos; and Greasley and Lamont (2011) collected 15 functions of music, as well. Ter Bogt et al. (2011) collected 19 functions of music from the literature and used confirmatory factor analysis to group them into five dimensions. In a clinical study with adolescents, Walker Kennedy (2010) found 47 functions of music that could be reduced to five dimensions.

By way of summary, extant empirical studies have used either an open approach—trying to capture the variety of musical functions in the course of surveys or questionnaire studies—or predefined collections of functions as they resulted from specific theoretical approaches or from literature research. These different approaches have led to quite heterogeneous collections of possible musical functions—from only few functions posited by a specific hypothesis, to long lists arising from open surveys. Moreover, although the many attempts to distill the functions of music to fewer dimensions have produced some points of agreement, the overall picture remains unclear.

### The structure among the functions of music

With each successive study of musical functions, the aggregate list of potential uses has grown longer. Questionnaire studies, in particular, have led to the proliferation of possible ways in which music may be relevant in people's lives. Even if one sidesteps the question of possible evolutionary origins, the multitude of hundreds of proposed functions raises the question of whether these might not be distilled to a smaller set of basic dimensions.

As noted earlier, previous research appears to converge on four dimensions: *social functions* (such as the expression of one's identity or personality), *emotional functions* (such as the induction of positive feelings), cogni*tive or self-related functions* (such as escapism), and *arousal-related functions* (such as calming down or passing time). These four dimensions might well account for the basic ways in which people use music in their daily lives.

Notice that cluster analysis and PCA/factor analysis presume that the research begins with a range of variables that ultimately capture all of the factors or dimensions pertaining to the phenomenon under consideration. The omission of even a single variable can theoretically lead to incomplete results if that variable proves to share little variance in common with the other variables. For example, in studying the factors that contribute to a person's height, the failure to include a variable related to developmental nutrition will led to deceptive results; one might wrongly conclude that only genetic factors are important. The validity of these analyses depends, in part, on including a sufficient range of variables so that all of the pertinent factors or dimensions are likely to emerge.

Accordingly, we propose to address the question of musical functions anew, starting with the most comprehensive list yet of potential music-related functions. In addition, we will aim to recruit a sample of participants covering all age groups, a wide range of socio-economic backgrounds, and pursue our analysis without biasing the materials to any specific theory.

## Fundamental functions of music—a comprehensive empirical study

The large number of functions of music that research has identified during the last decades has raised the question of a potential underlying structure: Are there functions that are more fundamental and are there others that can be subsumed under the fundamental ones? And if so, how many fundamental functions are there? As we have outlined above, many scientists have been in search of basic distinct dimensions among the functions of music. They have used statistical methods that help uncover such dimensions among a large number of variables: factor analyses or cluster analyses.

However, as we have also seen, the approaches and methods have been as different as the various functions suggested. For instance, some scholars have focused exclusively on the social functions of music while others have been interested in only the emotional ones; some used only adolescent participants while others consulted only older people. Thus, these researchers arrived at different categorizations according to their particular approach. To date, there is still no conclusive categorization of the functions of music into distinct dimensions, which makes psychological studies that rely on the use of music and its effects on cognition, emotion, and behavior still difficult (see also Stefanija, 2007). Although there exist some theoretically driven claims about what fundamental dimensions there might be (Tarrant et al., 2000; Laiho, 2004; Schubert, 2009; Lonsdale and North, 2011), there has been no large-scale empirical study that analyzed the number and nature of distinct dimensions using the broad range of *all* potential musical functions—known so far—all at once.

We sought to remedy this deficiency by assembling an exhaustive list of the functions of music that have been identified in past research and putting them together in one questionnaire study. Based on the research reviewed in the first part of this study, we identified more than 500 items concerned with musical use or function. Specifically, we assembled an aggregate list of all the questions and statements encountered in the reviewed research that were either theoretically derived or used in empirical studies. Of course, many of the items are similar, analogous, or true duplicates. After eliminating or combining redundant items, we settled on a list of 129 distinct items. All of the items were phrased as statements in the form “I listen to music because … ” The complete list of items is given in Table A3, together with their German versions as used in our study.

### Method

Participants were asked to rate how strongly they agreed with each item-statement on a scale from 0 (*not at all*) to 6 (*fully agree*). When responding to items, participants were instructed to think of any style of music and of any situation in which they would listen to music. In order to obtain a sample that was heterogeneous with regard to age and socioeconomic background, we distributed flyers promoting the Internet link to our study in a local electronics superstore. Recruitment of participants was further pursued via some mailing lists of German universities, students from comprehensive schools, and members of a local choir. As an incentive, respondents got the chance to win a tablet computer. A total of 834 people completed the survey. Respondents ranged from 8 to 85 years of age (*M* = 26, *SD* = 10.4, 57% female).

Notice that in carrying out such a survey, we are assuming that participants have relatively accurate introspective access to their own motivations in pursuing particular musical behaviors, and that they are able to accurately recall the appropriate experiences. Of course, there exists considerable empirical research casting doubt on the accuracy of motivational introspection in self-report tasks (e.g., Wilson, 2002; Hirstein, 2005; Fine, 2006). These caveats notwithstanding, in light of the limited options for gathering pertinent empirical data, we nevertheless chose to pursue a survey-based approach.

### Results

Principal component analysis revealed three *distinct dimensions* behind the 129 items (accounting for about 40% of the variance), based on the scree plot. This solution was consistent over age groups and genders. The first dimension (eigenvalue: 15.2%) includes statements about self-related thoughts (e.g., music helps me think about myself), emotions and sentiments (e.g., music conveys feelings), absorption (e.g., music distracts my mind from the outside world), escapism (e.g., music makes me forget about reality), coping (e.g., music makes me believe I'm better able to cope with my worries), solace (e.g., music gives comfort to me when I'm sad), and meaning (e.g., music adds meaning to my life). It appears that this dimension expresses a very private relationship with music listening. Music helps people think about who they are, who they would like to be, and how to cut their own path. We suggest labeling this dimension *self-awareness*. The second dimension (eigenvalue: 13.7%) includes statements about social bonding and affiliation (e.g., music helps me show that I belong to a given social group; music makes me feel connected to my friends; music tells me how other people think). People can use music to feel close to their friends, to express their identity and values to others, and to gather information about their social environment. We suggest labeling this dimension *social relatedness*. The third dimension (eigenvalue: 10.2%) includes statements about the use of music as background entertainment and diversion (e.g., music is a great pastime; music can take my mind off things) and as a means to get into a positive mood and regulate one's physiological arousal (e.g., music can make me cheerful; music helps me relax; music makes me more alert). We suggest labeling this dimension *arousal and mood regulation*. All factor loadings are reported in Table A3.

**Figure 1:**
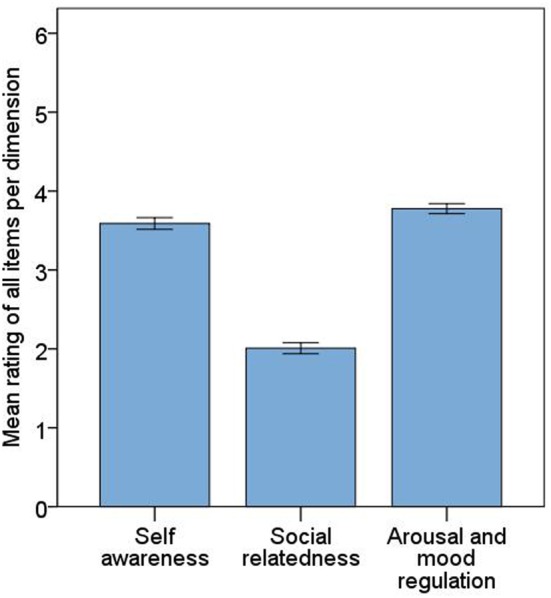
**The three distinct dimensions emerging from 129 reasons for listening to music**. Error bars are 95% confidence intervals. Self-awareness: *M* = 3.59 (*SE* = 0.037); social relatedness: *M* = 2.01 (*SE* = 0.035); arousal and mood regulation: *M* = 3.78 (*SE* = 0.032).

In order to analyze the relative significance of the three derived dimensions for the listeners, we averaged the ratings for all items contained in each dimension (see Figure 1). Arousal and mood regulation proved to be the most important dimension of music listening closely followed by self-awareness. These two dimensions appear to represent the two most potent reasons offered by people to explain why they listen to music, whereas social relatedness seems to be a relatively less important reason (ranging below the scale mean). This pattern was consistent across genders, socioeconomic backgrounds, and age groups. All differences between the three dimensions are significant (all *p*s < 0.001). The reliability indices (Cronbach's α) for the three dimensions are α = 0.97 for the first, α = 0.96 for the second, and α = 0.92 for the third dimension.

## General discussion

Since the earliest writing on the psychology of music, researchers have been concerned with the many ways in which people use music in their lives. In the first part of this paper, we reviewed literature spanning psychological, musicological, biological, and anthropological perspectives on musical function. The picture that emerged from our review was somewhat confusing. Surveying the literature from the past 50 years, we identified more than 500 purported functions for music. From this list, we assembled a somewhat catholic list of 129 non-redundant musical functions. We then tested the verisimilitude of these posited functions by collecting survey responses from a comparatively large sample. PCA revealed just three distinct dimensions: People listen to music to achieve *self-awareness*, *social relatedness*, and *arousal and mood regulation*. We propose calling these the Big Three of music listening.

In part one of our study we noted that several empirical studies suggest grouping musical functions according to four dimensions: cognitive, emotional, social/cultural, and physiological/arousal-related functions. This raises the question of how our three-dimensional result might be reconciled with the earlier work. We propose that there is a rather straightforward interpretation that allows the four-dimensional perspective to be understood within our three-dimensional result. Cognitive functions are captured by the first dimension (self-awareness); social/cultural functions are captured by the second dimensions (social relatedness); physiological/arousal-related functions are captured by the third dimension (arousal and mood regulation); and emotional functions are captured by the first and third dimensions (self-awareness + arousal and mood regulation). Notably—as can be seen with the items in Table A3—there is a dissociation of emotion-related and mood-related functions. Emotions clearly appear in the first dimension (e.g., music conveys feelings; music can lighten my mood; music helps me better understand my thoughts and emotions), indicating that they might play an important role in achieving self-awareness, probably in terms of identity formation and self-perception, respectively. However, the regulation of moods clearly appears in the third dimension (e.g., music makes me cheerful; music can enhance my mood; I'm less bored when I listen to music), suggesting that moods are not central issues pertaining to identity. Along with the maintenance of a pleasant level of physiological arousal, the maintenance of pleasant moods is an effect of music that might rather be utilized as a “background” strategy, that is, not requiring a deep or aware involvement in the music. The regulation of emotions, on the other side, could be a much more conscious strategy requiring deliberate attention and devotion to the music. Music psychology so far has not made a clear distinction between music-related moods and emotions; and the several conceptions of music-related affect remain contentious (see Hunter and Schellenberg, 2010). Our results appear to call for a clearer distinction between moods and emotions in music psychology research.

As noted earlier, a presumed evolutionary origin for music need not be reflected in modern responses to music. Nevertheless, it is plausible that continuities exist between modern responses and possible archaic functions. Hence, the functions apparent in our study may echo possible evolutionary functions. The three functional dimensions found in our study are compatible with nearly all of the ideas about the potential evolutionary origin of music mentioned in the introduction. The idea that music had evolved as a means for establishing and regulating social cohesion and communication is consistent with the second dimension. The idea of music satisfying the basic human concerns of anxiety avoidance and quest for meaning is consistent with the first dimension. And the notion that the basic function of music could have been to produce dissociation and pleasure in the listener is consistent with the third dimension.

In light of claims that music evolved primarily as a means for promoting social cohesion and communication—a position favored by many scholars—the results appear noteworthy. Seemingly, people today hardly listen to music for social reasons, but instead use it principally to relieve boredom, maintain a pleasant mood, and create a comfortable private space. Such a private mode of music listening might simply reflect a Western emphasis on individuality: self-acknowledgement and well-being appear to be more highly valued than social relationships and relatedness (see also Roberts and Foehr, 2008; Heye and Lamont, 2010).

The results of the present study may be of interest to psychologists who make use of music as a tool or stimulus in their research. The way people usually listen to music outside the laboratory will surely influence how they respond to musical stimuli in psychological experiments. For those researchers who make use of music in psychological studies, some attention should be paid to how music is used in everyday life. The three dimensions uncovered in this study can provide a parsimonious means to identify the value a person sets on each of three different types of music use. It is also conceivable that individual patterns of music use are related to personality traits, a conjecture which may warrant future research.

With regard to music cognition, the present results are especially relevant to studies about aesthetic preferences, style or genre preferences, and musical choice. Recent research suggests that musical functions play an important role in the formation and development of music preferences (e.g., Schäfer and Sedlmeier, 2009; Rentfrow et al., 2011). It will be one of the future tasks of music cognition research to investigate the dependence of music preference and music choice on the functional use of music in people's lives.

By way of summary, in a self-report study, we found that people appear to listen to music for three major reasons, two of which are substantially more important than the third: music offers a valued companion, helps provide a comfortable level of activation and a positive mood, whereas its social importance may have been overvalued.

### Conflict of interest statement

The authors declare that the research was conducted in the absence of any commercial or financial relationships that could be construed as a potential conflict of interest.

## Figures and Tables

**Table A1:**
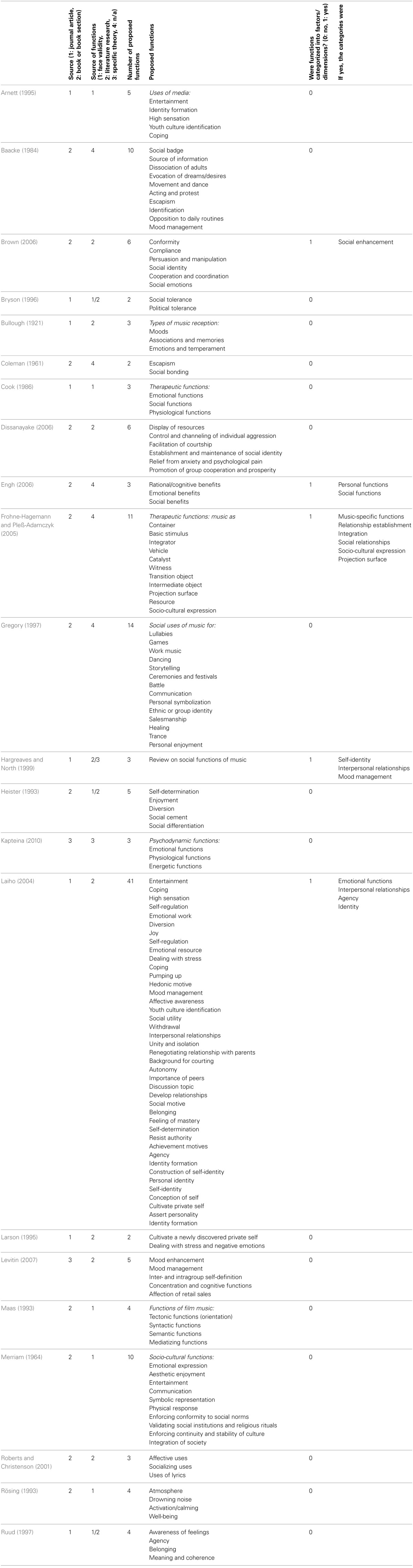
**Overview of theoretical contributions that have derived, proposed, or addressed more than one function or functional aspect of music listening**.

**Table A2:**
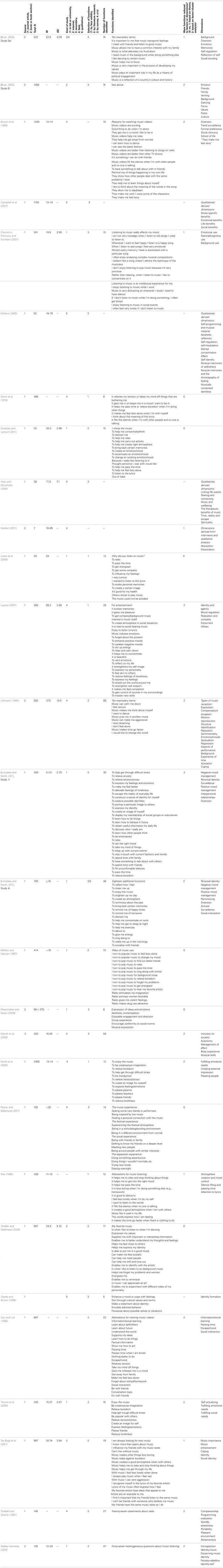
**Overview about empirical studies that have identified and/or investigated more than one function or functional aspect of music listening**.

*In some places, we could only provide exemplary functions because either the total number of functions was too large to be displayed here or not all functions were given in the original publications*.

## Appendix

**Table A3:** **The 129 statements referring to the functions of music exhaustively derived from past research, together with their means, standard deviations, and factor loadings (varimax rotated)**.

**I listen to music … [Ich höre Musik …]**	**Statistics**		**Factor loadings**		
	***M***	***SD***	**1**	**2**	**3**
Because it helps me think about myself.					
[weil ich dann gut über mich nachdenken kann.]	3.53	1.82	0.715		
Because it can lead my thoughts to somewhere else.					
[weil ich dann in Gedanken ganz weit weg sein kann.]	4.21	1.70	0.671		
Because it makes me believe I am better able to cope with my worries.					
[weil ich dann das Gefühl habe, mit meinen Sorgen besser fertig zu werden.]	3.37	1.87	0.668		
Because it helps me better understand my thoughts and emotions.					
[weil sie hilft, meine Gedanken und Gefühle besser zu verstehen.]	3.02	1.85	0.667		
Because it helps me think about my identity.					
[weil sie mir hilft, über meine Identität nachzudenken.]	2.87	1.94	0.665		
Because it is therapy for my soul.					
[weil sie eine Therapie für meine Seele ist.]	3.82	1.84	0.656		
Because it gives comfort to me when I'm sad.					
[weil sie mir Trost spendet, wenn ich traurig bin.]	4.10	1.73	0.645		
Because it makes me feel secure.					
[weil ich mich dann geborgen fühle.]	3.00	1.79	0.637		
Because it is a means to express myself.					
[weil sie mir eine Möglichkeit bietet, mich selbst auszudrücken]	3.39	1.89	0.632		
Because it helps me find my own way.					
[weil sie mir hilft, meinen Weg zu finden.]	3.07	1.85	0.625		
Because it mirrors my feelings and moods.					
[weil ich darin meine Gefühle und Stimmungen wiederfinde.]	4.92	1.31	0.610		
Because it conveys feelings.					
[weil sie Gefühle transportiert.]	4.80	1.38	0.608		
Because it expresses something that cannot be expressed in words.					
[weil sie etwas vermittelt, was sich in Worten nicht ausdrücken lässt.]	3.82	1.91	0.602		
Because it helps me learn about myself.					
[weil ich dadurch etwas über mich lernen kann.]	2.30	1.82	0.589		
Because it helps me be contemplative.					
[weil sie mir beim Nachdenken hilft.]	3.65	1.82	0.572		
Because it helps me escape from my daily routines.					
[weil ich dann aus dem grauen Alltag fliehen kann.]	3.62	1.85	0.567		
Because it often induces visual imagery.					
[weil ich dabei oft bildhafte Vorstellungen habe.]	3.88	1.72	0.564		
Because it can make me dream.					
[weil ich dabei träumen kann.]	4.49	1.50	0.562		
Because it distracts my mind from the outside world.					
[weil sie mich von der “Außenwelt” ablenkt.]	3.74	1.78	0.552		
Because it lets me forget the world around me.					
[weil ich dann die Welt um mich herum vergessen kann.]	4.48	1.57	0.551		
Because it makes me forget about reality.					
[weil sie mich die Realität vergessen lässt.]	3.30	1.91	0.544		
Because it puts fantastic images or stories in my head.					
[weil mir dann tolle Bilder oder Geschichten in den Kopf kommen.]	3.88	1.76	0.543		
Because it alleviates my inner tension.					
[weil das die Anspannung in mir verringert.]	3.85	1.55	0.542		
Because it helps me reminisce.					
[weil ich dabei in Erinnerungen schwelgen kann.]	4.24	1.64	0.532		
Because it gives me the energy I need for the day.					
[weil sie mir Energie für den Tag gibt.]	4.44	1.47	0.531		
Because I can recognize myself in the lyrics.					
[weil ich mich in den Texten wiederfinden kann.]	3.72	1.70	0.524		
Because it makes me feel somebody else feels the same as I do.					
[weil sie mir das Gefühl gibt, dass jemand anderes dasselbe fühlt wie ich.]	3.13	1.94	0.521		
Because it supports my ideas.					
[weil sie meine Ideen unterstützt.]	2.86	1.82	0.512		
Because it lets me be the way I am.					
[weil ich dadurch so sein kann, wie ich bin.]	3.66	1.89	0.508		
Because it enables me to experiment with different facets of my personality.					
[weil sie mir ermöglicht, mit verschiedenen Seiten meiner Persönlichkeit zu experimentieren.]	2.52	1.94	0.508		
Because it calms me.					
[weil sie mich beruhigt.]	4.32	1.37	0.501		
Because it adds meaning to my life.					
[weil sie mir Sinn im Leben gibt.]	2.24	2.03	0.496		
Because it can reduce my anxiety.					
[weil sie meine Angst reduzieren kann.]	2.51	1.93	0.493		
Because it makes me feel that I want to change the world.					
[weil ich dann das Gefühl bekomme, dass ich die Welt verändern möchte.]	2.34	1.96	0.489		
Because it is a means of venting my frustration.					
[weil sie eine Möglichkeit bietet, meine Frustration abzuladen.]	3.82	1.82	0.488		
Because it can reduce my stress.					
[weil sie meinen Stress reduziert.]	4.42	1.39	0.487		
Because it can make me feel less lonely.					
[weil ich mich dann weniger einsam fühle.]	2.93	1.92	0.486		
Because I like the bodily changes it evokes (changes of heartbeat, prickling, etc.)					
[weil ich die körperlichen Wirkungen mag (Veränderung des Herzschlags, Kribbeln auf der Haut usw.), die sie auslöst.]	3.33	2.02	0.483		
Because it gives me a way to let off steam.					
[weil ich dadurch Dampf ablassen kann.]	3.82	1.83	0.479		
Because it can lighten my mood.					
[weil sie meine Gefühle positiv beeinflussen kann.]	4.86	1.22	0.473		
Because it gives me pleasure.					
[weil sie Wohlgefallen auslöst.]	4.54	1.49	0.473		
Because it reminds me of certain periods of my life or past experiences.					
[weil sie mich an bestimmte Phasen meines Lebens bzw. an vergangene Ereignisse erinnert.]	4.62	1.51	0.471		
Because I just enjoy listening to music.					
[weil ich es einfach genieße, Musik zu hören.]	5.33	1.06	0.460		
Because it gives me intellectual stimulation.					
[weil es eine intellektuelle Stimulation für mich ist.]	2.94	1.90	0.434		
Because it gives me something that is mine alone.					
[weil ich dann etwas für mich alleine habe.]	2.58	2.00	0.418		
Because it gives me goose bumps.					
[weil ich dann Gänsehaut bekomme.]	3.16	1.91	0.416		
Because it addresses my sense of aesthetics.					
[weil sie meinen Sinn für Ästhetik anspricht.]	2.94	2.04	0.386		
Because it reminds me of a particular person.					
[weil sie mich an eine bestimmte Person erinnert.]	3.39	1.88	0.379		
Because it makes me feel my body.					
[weil ich dabei meinen Körper spüre.]	2.43	1.89	0.376		
Because I can enjoy it as art.					
[weil ich sie als Kunst genießen kann.]	3.63	1.93	0.358		
Because I want to play or sing it myself.					
[weil ich sie nachspielen oder nachsingen möchte.]	3.13	1.98	0.316		
Because it helps me show that I belong to a given social group.	1.30	1.62		0.726	
[weil ich damit zeigen kann, dass ich einer bestimmten sozialen Gruppe angehöre.]					
Because it makes me feel connected to all people who like the same kind of music.					
[weil ich mich dann allen Leuten zugehörig fühle, die solche Musik hören.]	1.68	1.71		0.686	
Because it makes me feel connected to my friends.					
[weil sie dazu führt, dass ich mich mit meinen Freunden verbunden fühle.]	2.02	1.73		0.671	
Because it provides me useful information for my everyday life.					
[weil ich dadurch nützliche Informationen für das alltägliche Leben sammeln kann.]	1.67	1.65		0.665	
Because it is a reason to meet my friends.					
[weil sie einen Grund dafür bietet, mit meinen Freunden zusammen zu sein.]	1.86	1.73		0.662	
Because it makes me feel connected to others.					
[weil ich mich durch sie mit anderen verbunden fühle.]	2.28	1.75		0.661	
Because it can help me meet other people.					
[weil ich dadurch neue Leute kennenlernen kann.]	2.15	1.78		0.661	
Because it helps me form friendships with people who have similar musical taste.					
[weil sie mir hilft, Freundschaften mit Personen zu schließen, die einen ähnlichen Musikgeschmack haben wie ich.]	2.17	1.82		0.660	
Because it tells me how other people think.					
[weil ich dann weiß, wie andere Leute denken.]	1.89	1.72		0.636	
Because I can learn something about other people.					
[weil ich dadurch etwas über andere lernen kann.]	2.49	1.76		0.629	
Because music is a social experience.					
[weil Musik eine Gruppenerfahrung ist.]	2.00	1.77		0.628	
Because it helps me develop social values.					
[weil Musik hilft, soziale Werte zu entwickeln.]	2.44	1.80		0.622	
Because I would like to identify with a particular music scene.					
[weil ich mich mit einer bestimmten Musikszene identifizieren möchte.]	1.75	1.83		0.608	
Because it helps me understand the world better.					
[weil ich dadurch die Welt besser verstehen kann.]	2.26	1.75		0.600	
Because it mirrors the history and culture of my country.					
[weil sie die Kultur und die Geschichte meines Landes widerspiegelt.]	1.15	1.58		0.588	
Because it can be a means to show political engagement.					
[weil sie ein wichtiges Mittel für mich ist, um politisches Engagement zu zeigen.]	1.00	1.50		0.582	
Because it helps me develop my personal values.					
[weil sie mir hilft, meine persönlichen Werte zu entwickeln.]	2.34	1.79		0.581	
Because it is a good way to express the uniqueness of our culture.					
[weil das ein gutes Mittel ist, um die Einzigartigkeit unserer Kultur auszudrücken.]	1.95	1.84		0.581	
Because I would like to take the artists/musicians as role models.					
[weil ich mir die Künstler/Musiker als Vorbild nehmen möchte.]	1.83	1.83		0.575	
Because it is something my friends like to do, as well.					
[weil das etwas ist, was meine Freundinnen und Freunde auch gerne tun.]	1.71	1.69		0.575	
Because it makes me feel connected to the world.					
[weil ich mich dann mit der Welt verbunden fühle.]	2.03	1.76		0.571	
Because it is something I can talk about with my friends.					
[weil ich dann etwas habe, worüber ich mich mit meinen Freundinnen und Freunden unterhalten kann.]	2.04	1.65		0.567	
Because I can be together with my family.					
[weil ich dabei mit meiner Familie zusammen sein kann.]	1.38	1.53		0.565	
Because it makes me belong.					
[weil ich somit “dazu gehöre.”]	.88	1.32		0.560	
Because my best friend and I can enthuse about it together.					
[weil meine beste Freundin/mein bester Freund und ich dann gemeinsam für etwas schwärmen können.]	1.62	1.69		0.543	
Because it can express my political attitudes.					
[weil sie meine politischen Überzeugungen ausdrücken kann.]	1.48	1.77		0.531	
Because my friends like the same music as I do.					
[weil sie auch meinen Freundinnen und Freunden gefällt.]	1.76	1.65		0.523	
Because when listening, I can imagine how the music would sound in a concert.					
[weil ich mir dabei vorstellen kann, wie die Musik wohl im Konzert wäre.]	2.54	1.97		0.496	
Because it is related to spirituality.					
[weil sie für mich eng mit Spiritualität verbunden ist.]	1.17	1.71		0.483	
Because I learn a lot about the world.					
[weil ich dadurch viel von der Welt erfahre.]	2.30	1.64		0.472	
Because I can identify with the musicians or bands.					
[weil ich mich dadurch mit einigen MusikerInnen oder Gruppen so gut identifizieren kann.]	2.58	1.79		0.465	
Because it supports my religious faith.					
[weil sie meinen Glauben unterstützt.]	1.22	1.83		0.448	
Because it has a supernatural meaning to me.					
[weil sie für mich eine übersinnliche Bedeutung hat.]	1.18	1.75		0.446	
Because I want to know what's going on in the music scene.					
[weil ich darüber Bescheid wissen will, was in der Musikszene gerade aktuell ist.]	1.97	1.82		0.429	
Because I want to find out something about the music.					
[weil ich etwas über die Musik herausfinden möchte.]	2.60	1.81		0.428	
Because it makes me let go of myself when I'm in company.					
[weil sie mir hilft, aus mir herauszugehen, wenn ich in Gesellschaft bin.]	2.81	1.84		0.422	
Because it contributes to my health.					
[weil sie zu meiner Gesundheit beiträgt.]	2.51	1.88		0.415	
Because it can soothe my physical pain.					
[weil sie meine körperlichen Beschwerden lindern kann.]	1.95	1.82		0.412	
Because you can learn something from the music.					
[weil man etwas dabei lernen kann.]	2.75	1.76		0.404	
Because I want to be informed about hits and trends.					
[weil ich mich über Hits und Trends informieren will.]	1.86	1.71		0.402	
Because it structures my everyday life.					
[weil sie meinem Alltag Struktur gibt.]	2.24	1.85		0.397	
Because I can get away from my family.					
[weil ich damit meiner Familie entkommen kann.]	1.31	1.73		0.378	
Because it is a means to share my memories with my friends.					
[weil sie eine Möglichkeit bietet, Erinnerungen mit Freunden zu teilen.]	3.40	1.81		0.375	
Because it makes me feel sexy.					
[weil ich mich dann sexy fühle.]	1.68	1.87		0.372	
Because I can learn about new pieces.					
[weil ich dabei neue Stücke kennenlernen kann.]	3.50	1.86		0.369	
Because I'm interested in the musicians and bands.					
[weil ich die MusikerInnen und Gruppen interessant finde.]	3.86	1.66		0.312	
Because it is a great pastime.	3.97	1.68			0.640
[weil sie ein prima Zeitvertreib ist.]					
Because it can take my mind off things.					
[weil sie mich ablenken kann.]	4.52	1.45			0.627
Because it prevents me from being bored while I do other things.					
[weil ich dann weniger gelangweilt bin, während ich etwas anderes tue.]	3.39	1.94			0.621
Because it makes time pass markedly faster.					
[weil dann die Zeit deutlich schneller vergeht.]	3.57	1.85			0.609
Because it enables me to kill time.					
[weil ich damit die Zeit totschlagen kann.]	2.64	1.99			0.598
Because I'm less bored then.					
[weil es dann nicht so langweilig ist.]	3.99	1.75			0.584
Because I need it in the background while I do other things.					
[weil ich sie im Hintergrund brauche, während ich etwas anderes tue.]	3.76	1.79			0.564
Because it makes me cheerful.					
[weil ich dann gute Laune bekomme.]	4.76	1.28			0.555
Because it can enhance my mood.					
[weil sie meine Stimmung verbessern kann.]	5.04	1.15			0.539
Because it fills the unpleasant silence when no one speaks.					
[weil Musik die unangenehme Stille füllt, wenn gerade niemand spricht.]	3.15	2.00			0.537
Because it helps me get up in the morning.					
[weil sie mir morgens hilft, wach zu werden.]	3.75	1.94			0.532
Because it helps me relax.					
[weil ich mich dann besser entspannen kann.]	4.84	1.18			0.520
Because it provides diversion.					
[weil sie für mich eine gute Abwechslung bietet.]	4.24	1.44			0.511
Because it puts me in the right mood for going out.					
[weil ich mich damit einstimmen kann, bevor ich ausgehe.]	3.69	2.05			0.508
Because it enhances my drive or my motivation for certain actions.					
[weil sie meinen Antrieb bzw. meine Motivation für bestimmte Tätigkeiten steigert.]	4.41	1.55			0.505
Because it is a good way to entertain myself.					
[weil das eine gute Art ist, mich selbst zu unterhalten.]	4.15	1.56			0.492
Because I take delight in doing so.					
[weil ich dabei Spaß habe.]	5.10	1.15			0.491
Because it makes me more alert.					
[weil ich dann wacher bin.]	3.32	1.72			0.477
Because it makes doing things seem effortless.					
[weil mir dann vieles lockerer von der Hand geht.]	4.40	1.40			0.464
Because it stimulates me.					
[weil sie mich animiert.]	3.96	1.63			0.441
Because I can dance to it.					
[weil ich dazu tanzen kann.]	3.53	2.04			0.436
Because it makes me feel fitter.					
[weil ich mich dann fitter fühle.]	3.37	1.81			0.432
Because it enables me to work off my aggression.					
[weil ich dabei meine Aggressionen abreagieren kann.]	3.48	2.02			0.427
Because it takes my mind off things.					
[weil sie mich auf andere Gedanken bringt.]	4.82	1.27			0.421
Because it provides a pleasant ambience for conversations.					
[weil sie eine angenehme Atmosphäre beim Gespräch schafft.]	3.17	1.72			0.416
Because music just fits into my life.					
[weil Musik einfach gut in mein Leben passt.]	4.90	1.37			0.403
Because it fits my sports.					
[weil es zu meinem Sport passt.]	2.62	2.16			0.400
Because working is easier with music.					
[weil ich dann besser arbeiten kann.]	3.43	1.83			0.389
Because it helps me fall asleep.					
[weil sie mir beim Einschlafen hilft.]	2.68	2.03			0.357
Because I can cuddle with my partner.					
[weil ich mit meinem Partner bzw. meiner Partnerin dabei gut kuscheln kann.]	2.50	1.85			0.354
Because I can sing or hum along.					
[weil ich dabei mitsingen oder mitsummen kann.]	3.91	1.80			0.346
Because I can try out new movements.					
[weil ich dann neue Bewegungen ausprobieren kann.]	1.83	1.83			0.337

*Dimension 1, self-awareness; Dimension 2, social relatedness; Dimension 3, arousal and mood regulation*.

## References

[B1] Arnett J. J. (1995). Adolescents' uses of media for self-socialisation. J. Youth Adolesc. 24, 519–533

[B2] Baacke D. (1984). Kommunikations-kultur der Jugend, in Medienpädagogik and Kommunikationskultur. Referate und Texte Nach Dem Ersten “Forum Kommunikationskultur,” ed de Haen I. (Frankfurt am Main: GEP), 37–53

[B3] Bartlett D. L. (1996). Physiological responses to music and sound stimuli, in Handbook of Music Psychology, 2nd Edn, ed Hodges D. A. (St. Louis, MO: MMB Music), 343–385

[B4] Bicknell J. (2007). Explaining strong emotional responses to music: sociality and intimacy. J. Conscious. Stud. 14, 5–23

[B5] Blood A. J. Zatorre R. J. (2001). Intensely pleasurable responses to music correlate with activity in brain regions implicated in reward and emotion. Proc. Natl. Acad. Sci. U.S.A 98, 11818–11823 10.1073/pnas.191355898 11573015 PMC58814

[B5a] Boehnke K. Münch T. (2003). Jugendsozialisation und Medien. Helfen Medien und Musik beim Erwachsenwerden? in Neue Medien im Alltag. Nutzung, Vernetzung, Interaktion, eds Keitel E. Boehnke K. Wenz K. (Lengerich: Pabst Science Publishers), 203–227

[B6] Boer D. (2009). Music Makes the People Come Together: Social Functions of Music Listening for Young People Across Cultures. Department of Psychology. Victoria University of Wellington, Wellington. Available online at: http://researcharchive.vuw.ac.nz/bitstream/handle/10063/1155/thesis.pdf?sequence=1

[B7] Brown J. D. Campbell K. Fischer L. (1986). American adolescents and music videos: why do they watch. Int. Commun. Gaz. 37, 19–32 10.1177/001654928603700104

[B8] Brown S. (2006). How does music work? Toward a pragmatics of musical communication, in Music and Manipulation: On the Social Uses and Social Control of Music, eds Brown S. Volgsten U. (New York, NY: Berghahn Books), 1–30

[B9] Bryson B. (1996). “Anything but heavy metal”: symbolic exclusion and musical dislikes. Am. Soc. Rev. 61, 884–899 10.2307/2096459

[B10] Bullough E. (1921). Recent work in experimental aesthetics. Br. J. Psychol. 12, 76–99

[B11] Campbell C. Connell S. Beegle A. P. (2007). Adolescents' expressed meanings of music in and out of school. J. Res. Music Educ. 55, 220–236

[B12] Chamorro-Premuzic T. Furnham A. (2007). Personality and music: can traits explain how people use music in everyday life. Br. J. Psychol. 98, 175–185 10.1348/000712606X111177 17456267

[B13] Coleman J. S. (1961). Psychological effects of the social system, in The Adolescents Society: The Social Life of the Teenager and its Impact on Education, ed Coleman J. S. (Oxford: Free Press of Glencoe), 220–243

[B14] Cook J. D. (1986). Music as an intervention in the oncology setting. Cancer Nurs. 9, 23–28 3518914

[B15] Darwin C. (1871). The Descent of Man, and Selection in Relation to Sex. London: John Murray

[B16] Darwin C. (1872). The Expression of the Emotions in Man and Animals. London: John Murray

[B17] DeNora T. (1999). Music as a technology of the self. Poetics 27, 31–56 10.1016/S0304-422X(99)00017-0

[B18] Dissanayake E. (2006). Ritual and ritualization: musical means of conveying and shaping emotion in humans and other animals, in Music and Manipulation: On the Social Uses and Social Control of Music, eds Brown S. Volgsten U. (New York, NY: Berghahn Books), 31–56

[B19] Dissanayake E. (2009). Root, leaf, blossom, or bole: concerning the origin and adaptive function of music, in Communicative Musicality: Exploring the Basis of Human Companionship, eds Malloch S. Trevarthen C. (New York, NY: Oxford University Press), 17–30

[B20] Engh M. (2006). Popstars als Marke: Identitätsorientiertes Marken-management für die Musikindustrielle Künstlerentwicklung und –Vermarktung. Wiesbaden: Deutscher Universitäts-Verlag

[B21] Fachner J. (2008). Musik und veränderte Bewusstseinszustände [Music and altered states of consciousness], in Musikpsychologie. Das neue Handbuch, eds Bruhn H. Kopiez R. Lehmann A. C. (Reinbek: Rowohlt), 594–612

[B22] Falk D. (2004a). Prelinguistic evolution in early hominins: whence motherese. Behav. Brain Sci. 27, 491–503 10.1017/S0140525X04000111 15773427

[B23] Falk D. (2004b). The “putting the baby down” hypothesis: bipedalism, babbling, and baby slings. Behav. Brain Sci. 27, 526–534 10.1017/S0140525X0448011X

[B24] Fine C. (2006). A Mind of Its Own: How Your Brain Distorts and Deceives. New York, NY: W.W. Norton

[B25a] Fitch W. T. (2006). The biology and evolution of music: a comparative perspective. Cognition 100, 173–215 10.1016/j.cognition.2005.11.009 16412411

[B26] Frith S. (1996). Performing Rites. On the Value of Popular Music. Oxford: Oxford University Press

[B27] Frohne-Hagemann I. Pleß-Adamczyk H. (2005). Indikation Musiktherapie bei psychischen Problemen im Kindes- und Jugendalter. Musiktherapeutische Diagnostik und Manual nach ICD-10. Göttingen: Vandenhoeck and Ruprecht

[B28] Gantz W. Gartenberg H. M. Pearson M. L. Schiller S. O. (1978). Gratifications and expectations associated with Pop music among adolescents. Pop. Music Soc. 6, 81–89 10.1080/03007767808591113

[B29] Greasley A. E. Lamont A. (2011). Exploring engagement with music in everyday life using experience sampling methodology. Music. Sci. 15, 45–71 10.1177/1029864910393417

[B30] Gregory A. H. (1997). The roles of music in society: the ethnomusicological perspective, in The Social Psychology of Music, eds Hargreaves D. J. North A. C. (New York, NY: Oxford University Press), 123–140

[B31] Hargreaves D. J. North A. C. (1999). The functions of music in everyday life: redefining the social in music psychology. Psychol. Music 27, 71–83 10.1177/0305735699271007

[B32] Hays T. Minichiello V. (2005). The meaning of music in the lives of older people: a qualitative study. Psychol. Music 33, 437–451 10.1177/030573560505616017062511

[B33] Heister H.-W. (1993). Stellenwert der Musik im gesellschaftlichen System, in Musikpsychologie. Ein Handbuch, eds Bruhn H Oerter R. Rösing H. (Reinbek: Rowohlt), 103–112

[B34] Herbert R. (2011). Everyday Music Listening: Absorption, Dissociation and Trancing. Farnham, Burlington: Ashgate Publishing Limited

[B35] Heye A. Lamont A. (2010). Mobile listening situations in everyday life: the use of MP3 players while travelling. Music. Sci. 14, 95–120

[B36] Hirstein W. (2005). Brain Fiction: Self-Deception and the Riddle of Confabulation. Cambridge, MA: MIT Press

[B37] Hunter P. G. Schellenberg E. G. (2010). Music and emotion, in Music Perception, Vol. 36, eds Jones M. R. Fay R. R. Popper A. N. (New York, NY: Springer), 129–164

[B38] Huron D. (2001). Is music an evolutionary adaptation?, in The Biological Foundations of Music, eds Zatorre R. J. Peretz I. (New York, NY: New York Academy of Sciences), 43–61 10.1111/j.1749-6632.2001.tb05724.x 11458859

[B39] Juslin P. N. Liljeström S. Västfjäll D. Barradas G. Silva A. (2008). An experience sampling study of emotional reactions to music: listener, music, and situation. Emotion 8, 668–683 10.1037/a0013505 18837617

[B40] Kapteina H. (2010). Was Geschieht, Wenn Wir Musik Hören. Fragmente Zur Psychologie Des Hörens. Available online at: http://www.musiktherapie.uni-siegen.de/kapteina/material/forschungsgebiete/neu_was_geschieht_wenn_wir_musik_hoeren.pdf

[B41] Laiho S. (2004). The psychological functions of music in adolescence. Nord. J. Music Ther. 13, 47–63 10.1080/08098130409478097

[B42] Larson R. (1995). Secrets in the bedroom: adolescents' private use of media. J. Youth Adolesc. 24, 535–550 10.1007/BF01537055

[B43] Laukka P. (2007). Uses of music and psychological well-being among the elderly. J. Happiness Stud. 8, 215–241 10.1007/s10902-006-9024-3

[B44] Lehmann A. C. (1994). Habituelle Und Situative Rezeptionsweisen Beim Musikhören. Eine Einstellungstheoretische Untersuchung. Frankfurt: Peter Lang

[B45] Levitin D. J. (2007). Life Soundtrack: The Uses of Music in Everyday Life. Montreal, QC: McGill University Available online at: http://levitin.mcgill.ca/pdf/LifeSoundtracks.pdf

[B46] Lonsdale A. J. North A. C. (2011). Why do we listen to music. a uses and gratifications analysis. Br. J. Psychol. 102, 108–134 10.1348/000712610X506831 21241288

[B47] Maas G. (1993). Filmmusik, in Musikpsychologie. Ein Handbuch, eds Bruhn H. Oerter R. Rösing H. (Reinbek: Rowohlt), 203–207

[B48] Maslow A. H. (1968). Toward a Psychology of Being. 2nd Edn New York, NY: Van Nostr and Company

[B49] McQuail D. Blumler J. G. Brown J. (1972). The television audience: a revised perspective, in Sociology of Mass Communication, ed McQuail D. (Middlesex: Penguin), 135–165

[B50] Melton G. W. Galician M. Lou. (1987). A sociological approach to the pop music phenomenon: radio and music video utilization for expectation, motivation and satisfaction. Pop. Music Soc. 11, 35–46 10.1080/03007768708591286

[B51] Merriam A. P. (1964). The Anthropology of Music. Evanston, IL: Northwestern University Press

[B52] Miller G. (2000). Evolution of human music through sexual selection, in The Origins of Music, eds Wallin N. L. Merker B. Brown S. (Cambridge: The MIT Press), 329–360

[B53] Misenhelter D. Kaiser K. (2008). Social functions of music in music education. J. Artistic Creat. Educ. 2, 61–74

[B54] Mithen S. (2006). The Singing Neanderthals: The Origins of Music, Language, Mind, and Body. Cambridge: Harvard University Press

[B55] Münch T. Bommersheim U. Müller-Bachmann E. (2005). Jugendliches Musikverhalten. Musikinvolvement, Nutzungsmotive und Musikpräferenzen, in Jugendsozialisation und Medien, eds Boehnke K. Münch T. (Lengerich: Pabst Science Publishers), 167–199

[B56] Nakamura J. Csikszentmihalyi M. (2009). Flow theory and research, in Oxford Handbook of Positive Psychology, 2nd Edn, eds Lopez S. J. Snyder C. R. (New York, NY: Oxford University Press), 195–206

[B57] Newberg A. D'Aquili E. Rause V. (2001). Why God Won't Go Away: Brain Science and the Biology of Belief. New York, NY: Ballantine Books

[B58] North A. C. Hargreaves D. J. O'Neill S. A. (2000). The importance of music to adolescents. Br. J. Educ. Psychol. 70, 255–272 10900782 10.1348/000709900158083

[B59] Packer J. Ballantyne J. (2011). The impact of music festival attendance on young people's psychological and social well-being. Psychol. Music 39, 164–181 10.1177/0305735610372611

[B60] Panksepp J. (1995). The emotional sources of “chills” induced by music. Music Percept. 13, 171–207

[B61] Panksepp J. Bernatzky G. (2002). Emotional sounds and the brain: the neuro-affective foundations of musical appreciation. Behav. Process. 60, 133–155 10.1016/S0376-635700080-3 12426066

[B62] Peretz I. (2006). The nature of music from a biological perspective. Cognition 100, 1–32 10.1016/j.cognition.2005.11.004 16487953

[B63] Rentfrow P. J. Goldberg L. R. Levitin D. J. (2011). The structure of musical preferences: a five-factor model. J. Pers. Soc. Psychol. 100, 1139–1157 21299309 10.1037/a0022406 PMC3138530

[B65] Roberts D. F. Christenson P. G. (2001). Popular music in childhood and adolescence, in Handbook of Children and the Media, eds Singer D. G. Singer J. L. (Thousand Oaks, CA: Sage Publications, Inc), 395–414

[B66] Roberts D. F. Foehr U. G. (2008). Trends in media use. Future Child. 18, 11–37 21338004 10.1353/foc.0.0000

[B67] Roe K. (1985). Swedish youth and music: listening patterns and motivations. Commun. Res. 12, 353–362 10.1177/009365085012003007

[B68] Rösing H. (1993). Musik im Alltag, in Musikpsychologie. Ein Handbuch, eds Bruhn H. Oerter R. Rösing H. (Reinbek: Rowohlt), 113–130

[B69] Ruud E. (1997). Music and the quality of life. Nord. J. Music Ther. 6, 86–97 10.1080/08098139709477902

[B70] Schäfer T. Sedlmeier P. (2009). From the functions of music to music preference. Psychol. Music 37, 279–300 10.1177/0305735608097247

[B71] Schäfer T. Sedlmeier P. (2010). What makes us like music. Determinants of music preference. Psychol. Aesthe. Creativity Arts 4, 223–234 10.1037/a0018374

[B72] Schubert E. (2009). The fundamental function of music. Music. Sci. 13, 63–81 10.1177/1029864909013002051

[B73] Steele J. R. Brown J. D. (1995). Adolescent room culture: studying the media in the context of everyday life. J. Youth Adolesc. 24, 551–576 10.1007/BF01537056

[B74] Stefanija L. (2007). Functions of music: a survey of research vocabularies. Muzikos funkcijos: tyrimø terminologijos apžvalga. (Lithuanian) 7, 6–17

[B75] Sun S. Lull J. (1986). The adolescent audience for music videos and why they watch. J. Commun. 36, 115–125 10.1111/j.1460-2466.1986.tb03043.x

[B76] Tarrant M. North A. C. Hargreaves D. J. (2000). English and American adolescents' reasons for listening to music. Psychol. Music 28, 166–173 10.1177/0305735600282005

[B77] Ter Bogt T. F. M. Mulder J. Raaijmakers Q. A. W. Gabhainn S. N. (2011). Moved by music: a typology of music listeners. Psychol.Music 39, 147–163 10.1177/0305735610370223

[B78] Troldahl V. C. Skolnik R. (1967). The meanings people have for radio today. J. Broadcast. 12, 57–67 10.1080/08838156709386226

[B79] Walker Kennedy S. (2010). An Exploration of Differences in Response to Music Related to Levels of Psychological Health in Adolescents. Toronto, ON: University of Toronto

[B79a] Wells A. Hakanen E. A. (1997). The emotional use of popular music by adolescents, in Mass Media and Society, eds Wells A. Hakanen E. A. (Greenwich: Ablex Publishing Corporation), 217–228

[B80] Wilson T. D. (2002). Strangers to Ourselves: Discovering the Adaptive Unconscious. Cambridge, MA: Harvard University Press

[B81] Zatorre R. J. Peretz I. (2001). The Biological Foundations of Music. New York, NY: New York Academy of Sciences

[B82] Zillmann D. (1988a). Mood management through communication choices. Am. Behav. Sci. 31, 327–341

[B83] Zillmann D. (1988b). Mood management: using entertainment to full advantage, in Communication, Social Cognition, and Affect, eds Donohew L. Sypher H. E. Higgins E. T. (Hillsdale, NJ: Lawrence Erlbaum Associates), 147–171

